# The Structure and Distribution of Benthic Communities on a Shallow Seamount (Cobb Seamount, Northeast Pacific Ocean)

**DOI:** 10.1371/journal.pone.0165513

**Published:** 2016-10-28

**Authors:** Cherisse Du Preez, Janelle M. R. Curtis, M. Elizabeth Clarke

**Affiliations:** 1 Institute for Ocean Sciences, Fisheries and Oceans Canada, Sidney, British Columbia, Canada; 2 Pacific Biological Station, Fisheries and Oceans Canada, Nanaimo, British Columbia, Canada; 3 Northwest Fisheries Science Center, National Marine Fisheries Service, NOAA, Seattle, Washington, United States of America; Department of Agriculture and Water Resources, AUSTRALIA

## Abstract

Partially owing to their isolation and remote distribution, research on seamounts is still in its infancy, with few comprehensive datasets and empirical evidence supporting or refuting prevailing ecological paradigms. As anthropogenic activity in the high seas increases, so does the need for better understanding of seamount ecosystems and factors that influence the distribution of sensitive benthic communities. This study used quantitative community analyses to detail the structure, diversity, and distribution of benthic mega-epifauna communities on Cobb Seamount, a shallow seamount in the Northeast Pacific Ocean. Underwater vehicles were used to visually survey the benthos and seafloor in ~1600 images (~5 m^2^ in size) between 34 and 1154 m depth. The analyses of 74 taxa from 11 phyla resulted in the identification of nine communities. Each community was typified by taxa considered to provide biological structure and/or be a primary producer. The majority of the community-defining taxa were either cold-water corals, sponges, or algae. Communities were generally distributed as bands encircling the seamount, and depth was consistently shown to be the strongest environmental proxy of the community-structuring processes. The remaining variability in community structure was partially explained by substrate type, rugosity, and slope. The study used environmental metrics, derived from ship-based multibeam bathymetry, to model the distribution of communities on the seamount. This model was successfully applied to map the distribution of communities on a 220 km^2^ region of Cobb Seamount. The results of the study support the paradigms that seamounts are diversity 'hotspots', that the majority of seamount communities are at risk to disturbance from bottom fishing, and that seamounts are refugia for biota, while refuting the idea that seamounts have high endemism.

## Introduction

A shallow seamount is a submarine mountain, usually volcanic in origin, whose summit rises more than 1000 m above the surrounding seafloor and protrudes into the euphotic zone (<200 m) [1]. Despite being important to commercial fisheries and of potential interest for industrial seabed mining [2], the ecology of these global marine features is largely understudied. The combination of accessibility, exploitation, and lack of baseline information makes shallow seamounts particularly vulnerable to human impact [3]. As states move forward with efforts to protect vulnerable marine ecosystems (VMEs) under international guidelines [3], shallow seamount management and conservation efforts will be at a disadvantage for not having baseline ecological information. More research has been conducted on deep seamounts (i.e., >800 m) but, in general, seamount ecology is still in its infancy with few paradigms withstanding scrutiny [1, 4, 5]. A research deficiency commonly mentioned is a lack of sufficient spatial coverage of biological data [5] to address ecological or management questions [6].

Few studies aim to resolve high-resolution community patterns on individual seamounts, and even fewer on shallow seamounts. At the scale of the individual seamount, community responses to depth are consistently the most obvious patterns that emerge, but other specific relationships and interactions are not well resolved [4]. For example, recent work on another Northeast Pacific seamount successfully resolved the vertical zonation of four distinct benthic megafaunal communities, but this was a deep seamount and the study resolution was relatively coarse (Davison Seamount summit depth: 1250 m; survey scale: hundreds of meters-squared [7]).

**Fig 1:**
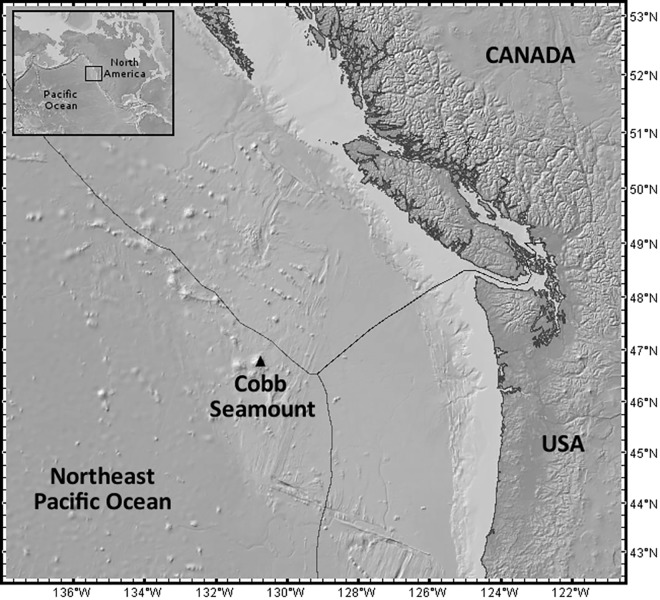
A regional map showing the location of Cobb Seamount (46° 44 N, 130° 48' W), approximately 500 km off the west coast of Canada and the USA, in the international waters of the Northeast Pacific Ocean. The exclusive economic zones are outlined. Map produced using www.geomapapp.org and the global multi-resolution topography synthesis [22].

The present study is focused on Cobb Seamount. Since its discovery in 1950, during an exploratory fishing program 500 km off the coast of Washington state, United States of America [8], Cobb Seamount has been the site of many oceanic expeditions (Fig 1). Research on this unusually shallow Northeast Pacific seamount has detailed its geology and geomorphology [9–12], oceanography [13, 14], fisheries [15, 16], and benthic ecology [17, 18] (summit depth: 24 m [18]). Cobb Seamount is regarded as a biological oasis amid the abyss, and is an important international fishing ground and a site for biodiversity conservation [12, 19]. Documented species include commercial fish species (e.g., *Anoplopoma fimbria* sablefish and *Sebastes* spp. rockfishes [18, 20]), and it was recently suggested Cobb Seamount may be refugia for mainland marine ecosystems [21]. Although the benthos of Cobb Seamount has been visually surveyed in the past (pinnacle top [17]; <180 m [18]; <700 m depth [11]), information on the benthic community structure, composition, species turnover, and distribution has been limited to qualitative observations of communities and their patterns of vertical zonation.

The purpose of the present study was to (1) resolve the community structure of the benthic mega-epifauna on a shallow seamount at a high-resolution (survey scale: meters-squared; resolution: centimetre); (2) investigate how these communities differ in composition; (3) examine the distribution of these communities in relation to seafloor environmental variation (depth and others); and (4) test the capability of using remotely-sensed environmental data to predict the spatial distribution of shallow seamount communities. Furthermore, (5) these findings on the structure, diversity, and distribution of benthic mega-epifauna communities on Cobb Seamount offer empirical evidence supporting or refuting several prevailing paradigms in seamount ecology [5].

## Methods and Materials

### Study Site

Cobb Seamount is located in the international waters of the Northeast Pacific Ocean (46° 44 N, 130° 48' W; part of the Cobb-Eickelberg Seamount chain; Fig 1). Spanning an area of 824 km^2^ (82400 hectares) and a depth range of 3 km, the seamount rises from the surrounding Cascadia Basin to a central pinnacle 24 m below the current sea level (ca. 12° slope [10]). It is located in the southward-flowing California Current but experiences a mean clockwise flow with evidence of a Taylor Proudman cap [14, 23]. Over the millennia since its formation, this guyot (flat-topped seamount) has been subject to significant sea-level changes, which, at times, caused it to be subaerial [10–12, 24, 25]. The subsidence history is still evident in the submarine ancient beaches, wave-cut terraces (at approximately 80, 150, 180, and 900 m depth), the eroded basalt pinnacle, and asymmetrical posteruptive slope modifications [10, 12]. The combination of volcanic, subaerial, intertidal, and submarine processes has created a mosaic of geological features on Cobb Seamount that provide a diversity of habitats for benthic organisms.

### Benthic Imagery Survey and Annotation

From July 21–26, 2012, a science team from Fisheries and Oceans Canada (DFO) and the United States National Oceanic and Atmospheric Administration (NOAA) conducted a benthic imagery ecological survey of Cobb Seamount from aboard the CCGS *John P*. *Tully*. The expedition fell under the jurisdiction of the United Convention on the Law of the Sea (UNCLOS). Under the Convention, all States have the right to conduct scientific research in the high seas [26]. The following is a summary of the non-invasive benthic imagery surveying and subsequent imagery annotation; for further details on either, refer to the expedition technical report [19] and species inventory [20].

Video and still photographs were collected from the summit and pinnacle (<220 m) using a remotely operated vehicle (ROV) and from the flanks (<1200 m) using a terrain-following autonomous underwater vehicle (AUV). The AUV could dive deeper, but was limited in dive duration (battery life) and surface interval time (recharging). For efficiency, the AUV was used to survey deeper areas and the tethered ROV was used to survey shallower areas during the AUV surface intervals. Dive sites were haphazardly selected to survey a range of environments for multiple expedition objectives.

The DFO Deep Ocean Engineering Phantom HD2+2 ROV was equipped with an 8 megapixel Cyclops digital still camera (C-Map Systems, Inc.) and a high-definition (HD) Mini Zeus video camera (1080i, Insite Pacific Inc.). The two cameras were mounted on a tilt mechanism and angled obliquely forward-facing (~45°). The video camera field of view (FOV) was lit by flood lights while the still camera FOV was lit by a separate strobe light (for enhanced image quality). Stills were taken every 15 seconds from between 0.5 to 1.5 m altitude. The camera systems were fitted with 10 cm parallel lasers to aid in species identification (sizing) and FOV calculations (as an estimate of the area visually surveyed). The NOAA SeaBED AUV was equipped with stereo still 5 megapixel, 12-bit dynamic range Prosilica GigE cameras mounted downward-facing, and the FOV was lit by strobe lights. Stills were taken every 10 seconds from approximately 4 m altitude. An onboard altimeter continuously recorded height above bottom, which was later analyzed to calculate the image FOV. For details on the NOAA AUV, refer to Clarke *et al*. [27]. Both submersibles were equipped with onboard navigational systems to record continuous coordinate and depth data.

**Fig 2:**
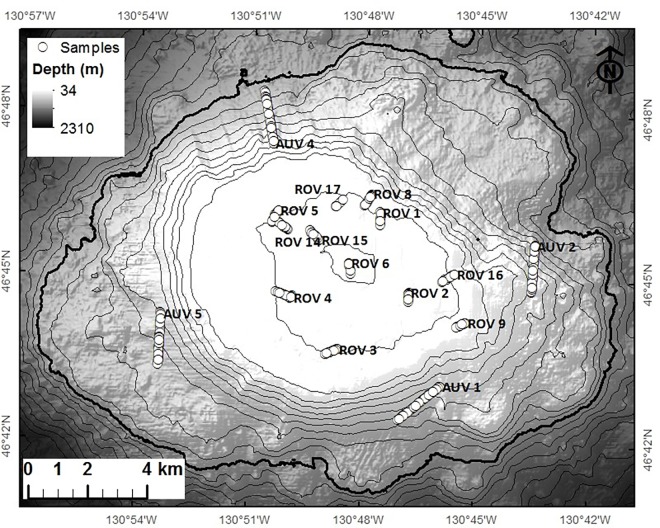
The location of submersible-collected images (circles) on Cobb Seamount. The imagery of the shallow seamount summit (<211 m) was collected using a remotely operated vehicle (ROV) while the imagery of the deeper flanks was collected using an autonomous underwater vehicle (AUV). Point locations of the images analysed are overlaid on shaded multibeam bathymetry where contour lines represent 100 m depth intervals.

During 16 submersible dives (12 ROV and 4 AUV), 13.5 km of benthic imagery surveying was completed yielding approximately 11 hours of ROV-collected video, 4000 ROV-collected still photographs, and 3000 AUV-collected still photographs (Fig 2). Names associated with dive sites correspond to those used in the cruise reports [19]. Images were collected along dive transects ranging from 350 to 1800 m in length. Depths surveyed ranged from 34 to 210 m and 473 to 1154 m (using the ROV and AUV respectively). Although attempts were made to survey the gap between 211 and 472 m, these dives had to be aborted because of technical issues.

The imagery was annotated using Video Miner (versions 2.1.3 and 2.1.4; a custom DFO image annotation software) and georeferenced using the submersible navigation databases. Annotated ecological data included the presence-absence of all resolvable (>0.5 cm) benthic organisms to the lowest taxonomic level possible with confidence (cf. [28]). Count data were obtained for a subset of taxa with individuals that were consistently discernible, which excluded encrusting colonial organisms, and organisms that were too abundant or too small in the images to confidently be resolved [19]. The subsequent clustering analyses could use both continuous and categorical data, but preliminary analyses combining the two data types ('presence-absence' and 'density' for a subset of taxa) did not alter results. A study in progress, focusing on VME indicator species (i.e., fragile habitat-forming species vulnerable to fishing disturbance [3]), will use the subset of count data (i.e., counts of mainly large, discrete cold-water corals and erect sponges). When an organism could not be identified to species, the lowest taxonomic level was provided followed by “sp.”. With limited voucher specimens collected, annotators drew on a compilation of published species checklists from Cobb Seamount, expert knowledge, and a range of taxonomic references to identify organisms. Nomenclature was based on the World Register of Marine Species database [29]. Annotated environmental data included the identification of the two dominant substrate types, where categories were defined as: Bedrock-Boulder, Bedrock-Gravel, Bedrock-Sand, Bedrock-Biological debris (e.g., coral rubble), Boulder-Gravel, Boulder-Sand, Gravel-Gravel, Gravel-Sand, and Sand-Biological debris. Annotated metadata included descriptions of the image quality, location relative to the seafloor, survey mode, and FOV. The metadata were used to quality control data included in the subsequent analyses. FOV measurements and distance transited (from navigation database) were used to estimate the area visually surveyed.

All AUV-collected still photographs, of approximately 5 m^2^, were analyzed at the centimeter scale. The ROV video was annotated for larger fauna in 10 second intervals to in effect visually survey ‘images’ of approximately 5 m^2^. A high-resolution ROV digital still image from each 10 second interval was analysed at the centimetre scale for smaller fauna undetectable in the video using a randomly selected 40 cm by 40 cm digital-overlay ‘quadrat’. The two-part ROV analysis was used to mitigate differences in equipment, set up, and capability between the two submersible types, facilitating comparability by matching the resolution and area visually surveyed (although we acknowledge that the two datasets may still be inherently different owing to the different submersibles used).

From the benthic imagery, 144 taxa were identified and recorded [20]. This size-based community subsample contained fauna of commercial value (e.g., groundfish) [19] as well as VME indicator species [30]. No single sampling method can survey all fauna and image surveys, such as the present study, are limited to resolvable, visible organisms (e.g., no infauna or microfauna). As such, the subset of fauna sampled is interpreted as representative only of the mega-epifaunal component of the community. Rare taxa—occurring in <1% of all images—and cryptic or small taxa that were inconsistently resolvable were removed from the analysed dataset. Samples (i.e., images) with <2 taxa present were also removed. To avoid overlapping and insure independence between consecutive images, the minimum nearest neighbor of an image was set at 5 meters (av. distance: >10 m). In total, data on 74 taxa from 1631 images (414 ROV and 1217 AUV) were retained for the community analyses.

### Community Analysis

To examine community structure, a two-step algorithm was used on a log-likelihood distance measure matrix of presence-absence untransformed data in PASW Statistics 18. The SPSS TwoStep Cluster Component analysis included sequentially creating many small sub-clusters of samples (i.e., images) based on ecological distance, and clustering the sub-clusters into the automatically-determined optimal number of clusters. The TwoStep Cluster Component was used because it is capable of automatically finding the optimal number of clusters, it outputs detailed summary statistics for each final cluster, and it can use both continues and categorical variables (used to assess the merit of including the partial count dataset). The automatically-determined optimal number of clusters was based on the distance between the two closest clusters in each hierarchical clustering stage and the comparison of a range of solutions using the Schwarz's Bayesian Criterion (BIC). Analysis outputs included a cluster assignment for each sample, the frequency of taxa occurrence in each cluster of samples, and a rank order of importance for each taxa in generating each cluster, which enables detailed investigation into the taxonomic composition of each cluster (i.e., community). The TwoStep Cluster Component does not generate a cluster diagram but illustrations of the (dis)similarities between clusters was generated using ordinations (see below). During the analysis, the input data sequence was randomized to avoid a collection order bias in the sequential analysis. Separate analyses were run for the two different survey methods (i.e., the shallower ROV and deeper AUV images).

The number of taxa (taxon richness) in each community, and the number of unique taxa between a pair of communities (where a low value means the two communities have similar compositions or little to no turnover) was also calculated as another means of contrasting community structure on the seamount.

### Environmental Analysis

The remotely-sensed gridded multibeam bathymetry was collected by NOAA in 2000 using a SeaBeam 2112 onboard the NOAA Ship RV *Ronald Brown* (survey RB0002). Despite the 12 year difference between the multibeam and the imagery expeditions, there was no evidence that a major landscape changing event had occurred, and it was determined the multibeam data still reflected the present bathymetry on Cobb Seamount in 2012. Four environmental datasets were derived from multibeam bathymetry of Cobb Seamount (20 m cell size raster): depth (in meters), slope (in degrees), and small- and large-scale metrics of roughness (complexity), specifically arc-chord ratio (ACR) rugosity at 4000 m^2^ and 4 km^2^ [31]. These two scales represent the smallest and largest areas that could be geoprocessed for multi-cell rugosity, given the resolution of the bathymetric data and the spatial distribution of the images. ACR rugosity was specifically used (over other complexity metrics) because it is decoupled from slope [31]. All spatial analyses and value extractions (to point image samples) were executed in ESRIArcMap 10.2.0.3348 using the Benthic Terrain Modeler [32] and the ACR tool [31]. Other variables originally derived from multibeam data (e.g., curvature and BPI indices) were found to be highly correlated with depth and removed from the analysis. The four variables included in the analysis represent non-correlated seafloor attributes, each known to influence species distributions [31].

To describe the seafloor environment of each community, the frequency of substrate types and the mean depth, slope, and small- and large-scale rugosity were determined for locations occupied by each identified community. To analyze the variance in these environmental variables between sites, Kruskal-Wallis one-way ANOVAs and pairwise comparisons using Mann-Whitney U tests were performed in PASW Statistics 18 (represented by box-plots). Owing to some environmental data not being collected or removed because of artifacts, the number of image locations used in all environmental analyses was further reduced from 1631 to 1464 (268 ROV and 1196 AUV).

### Community and Environmental Ordinations

To illustrate the ecological and environmental (dis)similarities between and within communities identified by the cluster analysis, ordinations were generated using non-metric multidimensional scaling (NMDS) in R using the "vegan" package [33, 34]. Ordinations were based on Bray-Curtis and Euclidean (dis)similarities between samples for the ecological and environmental data respectively (using "metaMDS"). Samples of the different communities were encircled by 95% confidence ellipses (using "ordiellipse"). Six ordinations were run in total: a set of ecological and environmental ordinations for all samples combined, the shallower ROV samples only, and the deeper AUV samples only. Interpretations of the ordinations were based on the shape and relative location of the ellipses. The concordance between sets of ordinations (i.e., multidimensional shape similarity) was examined to investigate whether the four environmental variables could explain the seamount community structure (using Procrustes rotation of two configurations and "PROTEST").

### Community Distribution Modelling

The benthos of Cobb Seamount was specifically investigated in relation to environmental data derived from remotely-sensed multibeam sonar to aid in and promote predictive modelling as a seamount management tool [6]. Cobb Seamount is only one of 382 known Northeast Pacific seamounts [35] and extrapolating its model could potentially provide predictions for other similar regional seamounts. To test predictive modelling on Cobb Seamount based on remotely-sensed environmental data (and to further investigate community distributions), a Random Forest model was generated using data for the community membership of each image and the environmental dataset for the four bathymetric environmental variables (in R using the "randomForest" package [36]). To match the resolution of the two datasets, the images were allocated to 20 m x 20 m cells and the most frequent community membership image data were retained (this reduced the image dataset to *n* = 579). Standard Random Forest model outputs included: a raster of the model predictions, a confusion matrix, an out-of -bag (OOB) error rate, and a metric of variable importance (i.e., mean decrease in accuracy). To provide further accuracy assessments of the model predictions and to indicate spatially explicit confidence, additional post hoc model performance analyses were included (in R): a 10-fold cross-validation of the area under the curve (AUC) analysis (the "pROC" package [37]; where an AUC value of 0.5 represents a model with no discrimination ability while an AUC of 1.0 represents a model with perfect discrimination [38, 39]), an indication of extrapolation (where one or more enivronmental variables were beyond the range of the data sampled), and an uncertainty analysis (i.e., the difference between bootstrap 5^th^ and 95^th^ percentile values; where uncertainity ranges from 0 to 1, or least to greatest uncertainty respectively). To produce a map with complete spatial coverage of the seamount, Kriging (in ArcMap) was used to interpolate the model predictions to cells with no environmental data (8.4% of the total number of cells, mostly on the seamount summit). The majority of R script used was developed at a Species Distribution Modelling workshop [40].

## Results

From the Cobb Seamount benthic imagery, the presence and absence of 74 benthic taxa from 11 phyla were recorded from 1631 images. Images ranged from 34 to 1154 m water depths (with a gap between 211 and 472 m). Taxa with a high frequency of presence in shallow ROV images (34 to 210 m; *n* = 414) included: *Stylaster* spp. hydrocorals (51%), *Pagurus kennerlyi* hermit crabs (43%), *Laqueus californianus* lamp shells (36%), and *Protula pacifica* tubeworms (26%). Taxa with a high frequency of presence in deep AUV images (475 m to 1154 m; *n* = 1217) included: *Pannychia* cf *moseleyi* sea cucumber (43%), Chirostylidae sp. squat lobsters (35%), *Sebastolobus* spp. thornyheads (30%), and *Bathypathes* sp. corals (20%). Overall, the shallower ROV images recorded more taxa (45 compared to 35 taxa, and despite more images being analysed to determine the latter), higher abundances of solitary fauna, and higher coverage of colonial fauna than the deeper AUV images.

### Community Analysis

**Fig 3:**
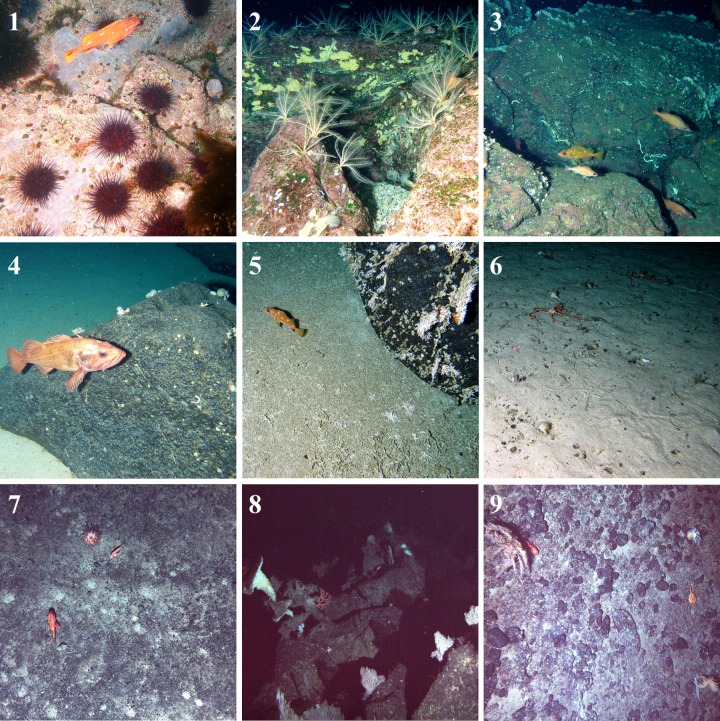
Representative photographs of the nine Cobb Seamount communities. (1) The hard substrate of the pinnacle carpeted in epifauna, including *Desmarestia viridis* macroalgae and *Mesocentrotus franciscanus* sea urchins. (2) Shallow, hard substrates with dense assemblages of *Florometra serratissima* crinoids on encrusting *Corallinales* spp. algae and *Halichondria panicea* sponge. (3) Hard substrates with a mix of Corallinales spp. and *Stylaster* spp. hydrocorals, supporting a rich assemblage of *Sebastes* spp. rockfishes. (4) Bare, hard substrate with small patches of the *Laqueus californianus* lamp shells and schools of the large *Sebastes* and *S*. *melanostictus* rockfishes (indistinguishable from images; recorded as one taxon). (5) A mix of hard and fine substrates, both covered in *Stylaster* spp. (living corals on hard, dead pieces and rubble on sand). (6) The flat, sandy edge of the seamount summit, with patches of *Ophiura sarsii* and *Asteronyx loveni* brittle stars, and *Pagurus kennerlyi* hermit crabs. (7) Sand and gravel on the seamount flanks, with sparse Actiniaria sp. anemones and *Sebastolobus* spp. thornyheads. (8) Exposed hard substrate on the deep flanks, with high-density Alcyonacea corals (e.g., *Paragorgia* sp.) and Hexactinellida sponges (e.g., *Pinulasma fistulosoma*). (9) Deep bedrock and gravel supporting a variety of Antipatharia spp. corals (e.g., *Lillipathes* cf *lillei* and *Bathypathes* sp.). Photo credit: DFO Pacific Biological Station ROV team, NOAA Northwest Fisheries Science Center and the Pacific Islands Fisheries Science Center AUV team.

Based on the ecological distance between samples, the cluster analyses assigned images to one of nine clusters. Images from the twelve ROV dives formed six clusters while images from the four AUV dives formed the other three clusters, or communities. The following paragraphs summarize the nine identified communities with regards to data obtained from the images, including: the frequency and importance ranking of taxa (Table 1), taxon richness and number of unique taxa (Table 2), statistics on the collection dives, and the frequency of substrate types (Table 3). The numbering of the community clusters reflects ecological similarity, where Community 1 is more similar to Community 2 than Community 3. Although they were analysed separately, the two sets of communities are sequentially numbered (1 to 6, and 7 to 9). To aid in relating community numbers to a community description, a name is also given to each of the communities (generally based on a defining characteristic). See Fig 2 for dive locations and Fig 3 for representative images of each community.

**Table 1:** The ecological summary statistics of the nine Cobb Seamount communities identified by the present study.

		Frequency of occurrence									Importance ranking								
Community		1	2	3	4	5	6	7	8	9	1	2	3	4	5	6	7	8	9
*n*		11	52	120	68	81	82	301	427	489	11	52	120	68	81	82	301	427	489
Ochrophyta																			
	*Desmarestia viridis*	46	0	0	0	0	0	0	0	0	7	35	37	37	35	43			
Rhodophyta																			
	Corallinales spp.	100	100	24	0	9	0	0	0	0	12	2	45	5	8	7			
Porifera																			
	cf *Acarnus erithacus*	55	8	0	0	0	0	0	0	0	8	16	26	23	21	29			
	*Auletta* sp.	0	0	3	2	1	0	0	0	0	40	30	25	44	45	37			
	Demospongiae sp.	0	37	1	2	0	0	0	0	0	32	5	22	22	15	23			
	*Farrea occa*	0	0	0	0	0	0	0	7	1							16	11	16
	*Halichondria panacea*	9	58	23	4	1	0	0	0	0	38	6	17	10	6	11			
	Rossellidae spp.	0	0	0	0	0	0	3	19	3							13	4	11
Cnidaria																			
	Actiniaria sp.	0	0	0	0	0	0	35	0	0							2	6	6
	*Anthoptilum* spp.	0	0	0	0	0	0	1	3	15							10	18	7
	*Bathypathes* sp.	0		0	0	0	0	12	2	41							12	3	2
	*Corynactis californica*	91	0	0	0	0	0	0	0	0	1	25	27	24	22	30			
	*Desmophyllum dianthus*	0	2	31	6	9	0	0	2	0	22	19	6	19	39	12	26	24	22
	*Gersemia* sp.	0	0	0	0	0	0	0	3	0							20	15	17
	*Halipteris willemoesi*	0	0	0	12	0	15	0	0	0	33	22	18	9	16	10	0	0	0
	*Heteropolypus ritteri*	0	0	0	0	0	0	5	7	27							7	12	5
	cf Hormathiidae sp.	0	0	0	0	0	0	0	4	0							21	13	15
	Isididae spp.	0	0	0	0	0	0	1	34	9							5	2	12
	*Lillipathes* cf *lillei*	0	0	0	0	0	0	2	6	38							4	5	1
	Pennatulacea sp.	0	0	0	0	0	0	4	3	1							23	33	23
	Primnoidae spp.	0	0	0	0	0	0	0	25	1							6	1	8
	*Stichopathes* sp.	0	0	0	0	0	0	1	7	3							15	17	28
	*Stomphia didemon*	0	39	0	0	0	0	0	0	0	34	4	19	17	17	25			
	*Stylaster* spp.	0	14	98	13	93	1	0	4	0	13	7	1	3	1	3	24	14	14
	*Swiftia simplex*	0	0	0	0	0	0	0	4	1							17	19	27
Annelida																			
	*Paradexiospira* sp.	9	0	32	12	1	1	0	0	0	43	14	4	43	10	17			
	*Phyllochaetopterus prolifica*	55	0	0	0	0	0	0	0	0	6	34	34	35	32	41			
	*Protula pacifica*	9	52	54	18	3	0	0	0	0	20	9	3	20	3	5			
	*Spiochaetopterus costarum*	9	12	3	54	1	50	0	0	0	27	20	8	2	4	4			
Arthropoda											^ ^	^ ^	^ ^	^ ^	^ ^	^ ^	^ ^	^ ^	^ ^
	*Chionocetes tanneri*	0	0	0	0	0	0	18	3	13							9	10	26
	Chirostylidae sp.	0	0	0	0	0	0	8	40	46							3	25	10
	*Chorilia longipes*	28	4	1	0	1	0	0	1	1	11	24	40	30	44	34	29	34	29
	*Lithodes cousei*	0	0	0	0	0	0	1	2	1							34	31	32
	*Pargurus kennerlyi*	0	19	22	32	77	72	0	0	0	15	12	9	18	2	6			
Mollusca																			
	*Calliostoma* spp.	18	0	0	0	0	0	0	0	0	2	26	28	36	23	31			
	*Crassadoma gigantean*	55	0	0	0	0	0	0	0	0	4	32	32	33	30	39			
	*Fusitriton oregonensis*	0	8	12	3	5	0	0	0	0	30	38	14	32	43	21			
	*Leptochiton rugatus*	55	0	0	0	0	0	0	0	0	5	33	33	34	31	40			
	Tritoniidae spp.	0	0	0	0	0	0	0	1	1							27	29	31
Brachiopoda																			
	*Laqueus californianus*	0	35	33	91	14	26	0	0	0	16	42	39	1	5	24			
Bryozoa																			
	cf *Reginella hippocrepis*	27	6	0	0	0	0	0	0	0	10	15	35	36	33	42			
Echinodermata																			
	*Ampheraster* sp.	0	0	0	0	0	0	3	1	2							30	28	35
	*Apostichopus leukothele*	0	14	23	12	9	1	0	0	0	21	44	10	42	34	15			
	*Asteronyx loveni*	0	0	0	6	0	45	0	0	0	26	17	11	29	9	2			
	Brisingidae sp.	0	0	0	0	0	0	0	0	8							14	21	9
	*Ceramaster patagon*	0	0	4	2	0	0	0	0	0	41	31	16	45	29	38			
	*Florometra serratissima*	27	64	0	2	1	1	1	4	0	17	1	12	13	13	19	28	20	18
	*Henricia sanguine*	0	6	6	2	1	0	0	0	0	37	23	24	39	37	27			
	*Hippasteria phrygiana*	0	0	0	2	0	0	1	2	0	45	41	41	14	42	45	33	26	19
	*Mesocentrotus franciscanus*	64	0	0	0	0	0	0	0	0	3	28	31	31	27	35			
	*Ophiopholis bakeri* ^a^	0	10	37	0	0	0	0	0	0	23	39	2	7	7	13			
	*Ophiura sarsii*	0	0	2	2	21	94	0	0	0	18	11	7	6	40	1			
	*Pannychia* cf *moseleyi*	0	0	0	0	0	0	33	50	43							11	23	34
	*Pseudarchaster* sp.	0	0	0	0	0	0	1	6	0							19	8	13
	*Psolus squamatus*	0	0	0	0	0	0	4	3	27							8	7	4
	*Pteraster* sp.	0	0	0	0	0	0	0	1	1							31	32	33
	*Rathbunaster californicus*	0	2	8	2	1	7	0	2	0	35	229	823	227	120	36	25	22	21
	*Stronglocentrotus pallidus*	0	0	6	0	1	0	0	0	0	39	27	13	28	41	33			
	*Thrissacanth* sp.	0	0	0	0	0	0	0	3	0							22	16	20
Chordata																			
	*Antimora microlpis*	0	0	0	0	0	0	0	1	0							35	30	30
	Ascidiacea sp. 1 (cream)	27	10	30	0	4	0	0	0	0	19	40	5	8	14	14			
	Ascidiacea sp. 2 (white)	18	8	3	0	1	0	0	0	0	14	18	42	21	38	28			
	cf *Coryphaenoides acrolepis*	0	0	0	0	0	0	7	4	3							18	35	24
	Cottidae sp.	0	12	8	3	6	26	0	0	0	24	43	38	15	25	9			
	*Embassichthys bathybius*	0	0	0	0	0	0	1	2	0							32	27	25
	*Sebastes aleutianus*^b^	0	2	3	15	1	0	0	0	0	36	37	43	4	26	26			
	*Sebastes alutus*	0	0	3	2	0	0	0	0	0	42	36	21	41	36	44			
	*Sebastes emphaeus*	0	27	14	15	1	0	0	0	0	25	10	30	25	11	16			
	*Sebastes helvomaculatus*	18	39	26	18	28	0	0	0	0	44	13	36	38	19	8			
	*Sebastes rosaceus*	36	2	2	2	0	0	0	0	0	9	45	44	40	24	32			
	*Sebastes variegatus*	0	0	10	12	3	0	0	0	0	31	21	20	11	28	22			
	*Sebastes wilsoni*	0	50	4	2	0	0	0	0	0	28	3	29	16	12	18			
	*Sebastes zacentrus*	0	23	13	0	3	0	0	0	0	29	8	15	12	18	20			
	*Sebatolobus alascanus*	0	0	0	0	0	0	85	19	7							1	9	3

For each community, the frequency of occurrence of benthic megafauna was calculated as the percentage of images in which the taxon was present (0% = absent, 100% = always present). The rank order of taxa importance for a community was assigned by the cluster analyses (1 = most important, no ranking = not included in the clustering process). The clustering of communities 1 to 6 and 7 to 9 considered 45 and 35 taxa respectively (which are therefore the lowest possible ranking values for those communities).

^a^ *O*. *bakeri* was sparsely observed and inconsistently resolvable in AUV images but was observed in easily dense, resolvable patches in the ROV images. *O*. *bakeri* was therefore included in the ROV dataset but excluded from the AUV dataset.

^b^ *S*. *aleutianus* and *S*. *melanostictus* are indistinguishable from images and these two species are recorded as one taxon.

**Table 2:** Taxon richness and number of unique taxa of the nine Cobb Seamount communities.

Community		1	2	3	4	5	6	7	8	9
Taxon richness		20	28	31	28	26	12	27	34	27
Number of unique taxa between communities:										
	1		24	31	34	26	26	43	50	43
	2			11	18	12	24	49	52	51
	3				11	7	25	54	57	56
	4					14	16	49	52	51
	5						18	47	50	49
	6							35	40	37
	7								9	8
	8									7
	Mean	35	30	32	31	28	28	37	40	38

The mean unique taxa measure is the average of the eight possible community pairings. Community 8, dominated by soft corals and glass sponges, has the highest taxon richness and number of unique taxa while community 6, associated with flat, sandy habitats, has the lowest of both.

**Table 3:** The frequency of each collection dive and each substrate type observed (as a percentage of the images that recorded each community), and the mean and range of the four environmental variables measured at the sample (i.e., image) locations for each community on Cobb Seamount.

Community		1	2	3	4	5	6	7	8	9
*n*		11	52	120	68	81	82	301	427	489
Collection dive (%)										
	AUV 1	0	0	0	0	0	0	49	3	5
	AUV 2	0	0	0	0	0	0	20	45	16
	AUV 4	0	0	0	0	0	0	7	44	32
	AUV 5	0	0	0	0	0	0	24	8	47
	ROV 1	0	0	0	0	0	55	0	0	0
	ROV 2	0	42	0	0	0	0	0	0	0
	ROV 3	0	0	12	12	11	12	0	0	0
	ROV 4	0	0	18	35	9	5	0	0	0
	ROV 5	0	0	25	9	12	0	0	0	0
	ROV 6	100	0	0	0	0	0	0	0	0
	ROV 8	0	0	1	32	4	0	0	0	0
	ROV 9	0	0	0	0	46	2	0	0	0
	ROV 14	0	0	30	4	17	0	0	0	0
	ROV 15	0	52	3	1	1	0	0	0	0
	ROV 16	0	0	0	1	0	26	0	0	0
	ROV 17	0	6	12	4	0	0	0	0	0
Substrate type (%)										
	Bedrock-Boulder	0	9	0	0	0	0	2	13	5
	Bedrock-Gravel	0	0	1	0	2	0	25	60	46
	Bedrock-Sand	82	39	9	5	12	0	0	0	0
	Bedrock-Biological debris	0	0	14	0	4	0	0	0	0
	Boulder-Gravel	0	3	0	0	0	0	1	1	0
	Boulder-Sand	0	48	65	63	25	3	6	2	3
	Boulder-Biological debris	0	0	9	0	11	0	0	0	0
	Gravel	0	0	0	0	0	0	13	8	12
	Gravel-Sand	18	0	0	26	7	94	52	15	33
	Sand-Biological debris	0	0	2	5	40	3	0	0	0
Environmental variable (mean, range)										
	Depth (m)	52, 34–89	142, 120–161	179, 155–206	196, 161–210	202, 167–212	198, 186–212	740, 536–1144	728, 473–1154	857, 548–1153
	Slope (°)	13, 1–31	5, 1–14	7, 1–21	4, 1–21	2, 0–8	2, 0–6	16, 3–51	19, 4–34	16, 3–34
	Rugosity at scale of 4,000 m^2^	1.07, 1.05–1.09	1.01, 1.00–1.01	1.01, 1.00–1.02	1.01, 1.00–1.03	1.01, 1.00–1.03	1.01, 1.00–1.02	1.07, 1.02–1.18	1.09, 1.03–1.18	1.07, 1.02–1.18
	Rugosity at scale of 4 km^2^	1.02, 1.01–1.02	1.02, 1.02–1.03	1.02, 1.02–1.04	1.03, 1.02–1.05	1.03, 1.02–1.04	1.04, 1.02–1.05	1.06, 1.04–1.09	1.07, 1.04–1.09	1.06, 1.04–1.09

See Fig 2 for dive locations.

**Community 1**—**Pinnacle Community**—had the densest assemblages of organisms observed on Cobb Seamount with structurally complex encrusting taxa that created a multilayer carpet over the pinnacle. Community 1 had the highest mean number of unique taxa (35) of the shallow-water communities (1 to 6), and dominant taxa of this community were not observed elsewhere on Cobb Seamount. For example, the only Ochrophyta (brown) macroalgae, *Desmarestia viridis* (46% frequency), was observed in this community. Of the 20 taxa observed, those with the highest frequency of presence were: Corallinales spp. algae (cf *Lithophyllum* spp. and cf *Lithothamnion* spp., 100%), *Corynactis californica* anemones (91%), *Mesocentrotus franciscanus* sea urchins (64%), cf *Acarnus erithacus* sponges, *Crassadoma gigantea* scallops, *Leptochiton rugatus* chitons, and *Phyllochaetopterus prolifica* tubeworms (all at 55%). Taxa with the highest importance in forming (i.e., defining) the community cluster were: *C*. *californica*, *Calliostoma* spp. topsnails (*C*. *annulatum and C*. *ligatum*, 18% frequency), *M*. *franciscanus*, and *C*. *gigantea*.

All Community 1 images (*n* = 11, 1% of all images) were observed on the seamount pinnacle during a single ROV dive from 34 to 90 m depth (ROV 1; lowest number of images assigned to one community cluster). Bedrock was a primary substrate in nearly all images (82%). Sand was always present (100%), but its coverage was nominal (a veneer or a pocket of sediment on the steep bedrock pinnacle). The most abundant substrate category was Bedrock-Sand (82%).

**Community 2—Crinoid Community**—was a rich assemblage of sessile, sedentary, and mobile taxa. The substrate was predominately encrusted with low-relief colonial organisms and sedentary filter-feeders with associated benthic *Sebastes* spp. rockfishes (from 6 species). This community had the highest frequency of *Sebastes* spp. of all the communities (88%; dominated by three medium-size species: *S*. *emphaeus*, *S*. *helvomaculatus*, and *S*. *zacentrus*). Of the 28 taxa observed, those with the highest frequency of presence were: Corallinales spp. (100%), *Florometra serratissima* crinoids (64%), *Halichondria panicea* sponges (58%), and *Protula pacifica* tubeworms (52%). Taxa with the highest importance in forming the community cluster were: *F*. *serratissima*, Corallinales spp., *Sebastes wilsoni* (50% frequency), *Stomphia didemon* anemones (39% frequency).

Community 2 images (*n* = 52, 3% of all images) were from the center of the summit plateau and observed during three ROV dives between 120 and 160 m depth (predominately ROV 15 and 2). The primary substrates in all Community 2 images were hard substrates (a mix of Bedrock and Boulders, 100%) and Sand (88%), and the two most abundant substrate categories were Boulder-Sand (48%) and Bedrock-Sand (39%).

**Community 3—Mixed Community**—had the highest taxon richness of the shallow-water communities and included nearly all taxa observed in the adjacent Communities 2, 4, and 5 (i.e., low number of unique taxa: 11, 11, and 7 respectively). The Mixed Community was dominated by *Stylaster* spp. and a diversity of encrusting, solitary life-forms (i.e., Annelida, Echinodermata, and Brachiopoda). Associated mobile fauna included a relatively high frequency and richness of echinoderms (i.e., brittle stars, sea stars, sea cucumbers, and sea urchins). Community 3 had the highest *Sebastes* spp. richness (8 to 9 species: *S*. *aleutianus* (and/or *S*. *melanostictus*), *S*. *alutus*, *S*. *emphaeus*, *S*. *helvomaculatus*, *S*. *rosaceus*, *S*. *variegatus*, *S*. *wilsoni*, and *S*. *zacentrus*). Of the 31 taxa observed, those with the highest frequency of presence were: *Stylaster* spp. (98%), *P*. *pacifica* (54%), *Ophiopholis bakeri* brittle stars (37%), and *L*. *californianus* (33%). Taxa with the highest importance in forming the community cluster were: *Stylaster* spp., *O*. *bakeri*, *P*. *pacifica*, and *Paradexiospira* sp. tubeworms (32% frequency).

Community 3 images (*n* = 120, 7% of all images) were from the western half of the summit plateau and observed during seven ROV dives between 155 and 205 m depth (predominately ROV 14, 5, 4, 3 and 17; near Community 4 images). Similar to Communities 2, 4, and 5, this community was found on a mosaic of Sand (76%) and hard substrates, but Community 3 had a greater frequency of hard substrates (98%) and the most abundant substrate category was Boulder-Sand (65%).

**Community 4—Bare Community**—was dominated by a mixture of Brachiopoda lamp shell patches on otherwise bare hard substrate and pockets of fine sediment with sessile Serpulidae worms (Annelida). *Sebastes* spp. were associated with the boulders and this community had the highest frequency of the large rockfish *S*. *aleutianus* and *S*. *melanostictus*. Of the 28 taxa observed, those with the highest frequency of presence were: *L*. *californianus* (91%), *Spiochaetopterus* cf *costarum* tubeworms (54%), *P*. *kennerlyi* (32%), and *S*. *helvomaculatus* (18%). Taxa with the highest importance in forming the community cluster were: *L*. *californianus*, *S*. cf *costarum*, *Stylaster* spp. (13% frequency), and *S*. *aleutianus* and *S*. *melanostictus* (15% frequency).

Community 4 images (*n* = 68, 4% of all images) were from the summit plateau and observed during eight ROV dives between 160 and 210 m depth (predominately ROV 4 and 8, and to a lesser extent ROV 3 and 5). This community was observed on a mosaic of Sand and hard substrate (Boulders). Sand was a primary substrate in all images (100%), and the most abundant substrate category was Boulder-Sand (63%).

**Community 5—Hydrocoral Community**—was dominated by living *Stylaster* spp. while over half the substrate was a mixture of dead hydrocorals, hydrocoral rubble, and fine sediment. Of the 26 taxa observed, those with the highest frequency of presence were: *Stylaster* spp. (93%), *P*. *kennerlyi* (77%), *S*. *helvomaculatus* (28%), and *Ophiura sarsii* brittle stars (21%). Taxa with the highest importance in forming the community cluster were: *Stylaster* spp., *P*. *kennerlyi*, *P*. *pacifica* (3% frequency), and *S*. cf *costarum* (1% frequency).

Community 5 images (*n* = 81, 5% of all images) were from the summit plateau, and observed during seven ROV dives between 170 and 210 m depth (predominately ROV 9, 14, 5, 3, and 4). This community was commonly observed on hard substrate (53%, mainly Boulders) but Sand was a primary substrate in nearly all images (84%). The single most abundant substrate category was Sand-Biological debris (40%) with Community 5 accounting for over half of all images with Biological debris as a primary substrate (52% of all images).

**Community 6—Sand Community**—was dominated by sparsely distributed brittle stars (Echinodermata) and had the lowest taxa richness and number of unique taxa of all the communities (12 and 28, respectively). Only one species of fish, a small Cottidae sp. sculpin, was recorded. Of the 12 taxa observed, those with the highest frequency of presence were: *O*. *sarsii* (94%), *P*. *kennerlyi* (72%), *S*. cf *costarum* (50%), and *Asteronyx loveni* brittle stars (45%). Taxa with the highest importance in forming the community cluster were: *O*. *sarsii*, *A*. *loveni*, *Stylaster* spp. (1% frequency), and *S*. cf *costarum*.

Community 6 images (*n* = 82, 5% of all images) were from the summit plateau and observed during five ROV dives between 190 and 210 m depth (predominately ROV 1, 16, and 3). Community 6 images showed little variation in substrate. Sand was a primary substrate in all images (100%) with almost no hard substrate (3%). The single most abundant substrate category was Gravel-Sand (94%; although it was mostly sand).

**Community 7—Anemone Community**—was largely barren, generally lacking structure-forming organisms except deep-sea anemones. Of the 27 taxa observed, those with the highest frequency of presence were: *Sebastolobus* spp. (85%), unidentified Actiniaria sp. anemones (35%), *P*. cf *moseleyi* (33%), and Chirostylidae sp. (18%). Taxa with the highest importance in forming the community cluster were: *Sebastolobus* spp., Actiniaria sp., Chirostylidae sp., and *Lillipathes* cf *lillei* corals (2% frequency).

Community 7 images (*n* = 301, 19% of all images) were from the seamount flanks and observed during the four AUV dives between 540 and 1140 m depth (majority from AUV 1). Gravel was a primary substrate in the majority of Community 7 images (91%), and only a minority of images had hard substrate (only 34%). The most abundant substrate category was Gravel-Sand (52%).

**Community 8—Soft coral Community**—had the highest taxa richness and number of unique taxa of the nine communities (34 and 40, respectively), and the highest frequency and occurrence of Alcyonacea (soft) corals and Hexactinellida (glass) sponges. Over two-thirds of images contained corals or glass sponges. Of the 34 taxa observed, those with the highest frequency of presence were: *P*. cf *moseleyi* (50%), Chirostylidae sp. (40%), Isididae spp. corals (*Isidella* sp., *Keratoisis* sp. and *Lepidisis* sp.; 34%), and Primnoidae spp. corals (*Plumarella superba* and *Primnoa* cf *pacifica*; 25%). Taxa with the highest importance in forming the community cluster were: Primnoidae spp., Isididae spp., *Bathypathes* sp. (2% frequency), and Rossellidae spp. sponges (*Acanthascus* spp., *Rhabdocalyptus* spp., *Staurocalyptus* spp. and *Bathydorus* sp.; 20% frequency). It should be noted that on occasion *O*. *bakeri* was observed but, owing to an inconsistency in ability to resolve its presence-absence, records of this taxon were excluded from the AUV dataset.

Community 8 images (*n* = 427, 26% of all images) were from the seamount flanks and observed during the four AUV dives between 470 and 1150 m depth (majority from AUV 2 and 4). Gravel was a primary substrate (84%) and Bedrock-Gravel was the most abundant substrate category (60%).

**Community 9—Black coral Community**—had the highest frequency of Antipatharia (black) corals with two-thirds of images containing at least one of the three observed species. Alcyonacea corals were also present but at a lower frequency. Mobile invertebrates (Arthropoda and Echinodermata) were abundant but fish (Chordata) were relatively infrequent. Of the 27 taxa observed, those with the highest frequency of presence were: Chirostylidae sp. (46%), *P*. cf *moseleyi* (43%), *Bathypathes* sp. (41%), and *L*. cf *lillei* (38%). Taxa with the highest importance in forming the community cluster were (high to low): *L*. *lillei*, *Bathypathes* sp., *Sebastolobus* spp. (7% frequency), and *Psolus squamatus* sea cucumbers (27% frequency).

Community 9 images (*n* = 489, 30% of all images) were from the seamount flanks and observed during the four AUV dives between 550 and 1150 m depth (majority from AUV 5 and 4; highest number of images assigned to one community cluster). Gravel was a primary substrate in nearly all images (92%) but over half also included a hard substrate (Bedrock or Boulder, 54%). The single most abundant substrate category was Bedrock-Gravel (46%).

#### Environmental Analysis

**Fig 4:**
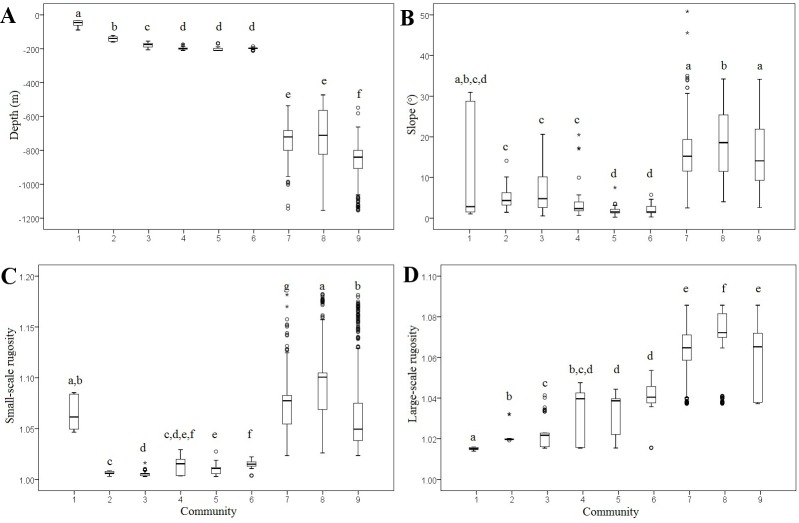
The environmental data (depth, slope, and small- and large-scale ACR rugosity) at the image locations for each of the nine Cobb Seamount communities. The boxes represent the inter-quartile interval, circles represent outliers, stars represent extreme outliers, and horizontal lines represent the median. The superscript letters represent the output of the Mann-Whitney U tests, where community locations that are significantly different to each other do not share a common letter.

Communities 1 to 6 were generally associated with shallow seafloor with a low-degree of slope and rugosity, while Communities 7 to 9 were generally associated with deep seafloor with a high degree of slope and rugosity. However, small- and large-scale rugosity and slope either had little to no correlation with depth (Spearman correlation: *p* = 0.837, *r* = 0.005; *p* < 0.001, *r* = 0.056; *p* < 0.001, *r* = 0.161; respectively). Using variance analyses, the locations where the nine communities were observed were shown to differ in environmental variables: depth, slope, and small- and large-scale ACR rugosity (Fig 4). For all four environmental variables the median for the locations of at least one community was significantly different from another (Kruskal-Wallis one-way ANOVA: *n* = 1464, *p* <<0.001). Pairwise comparisons for depth yielded the highest number of significant differences between community locations (88% of 36 Mann-Whitney U tests), followed by small-scale rugosity (83%), large-scale rugosity (81%), and slope (64%; Fig 4). There were only five cases where an environmental variable for a community location was unique (significantly different from every other community location): the depth at locations for Communities 1, 7, 8, and 9; and the fine- and large-scale rugosity locations for Community 2.

#### Community and Environmental Ordinations

**Fig 5:**
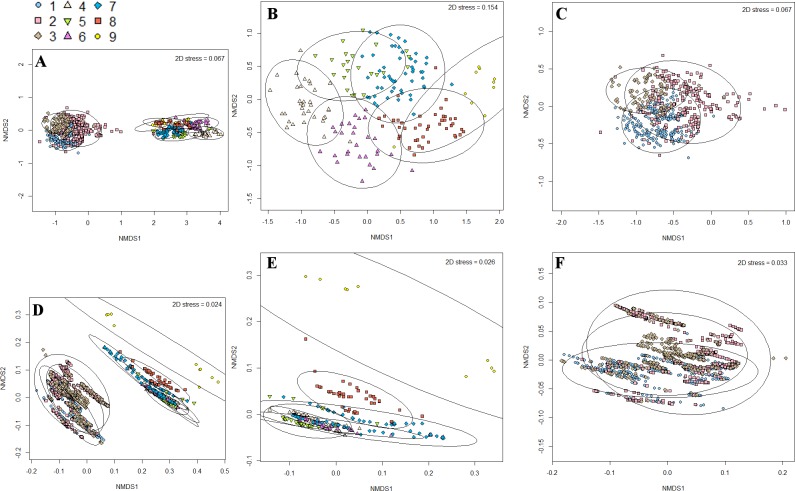
Non-metric multidimensional scaling (NMDS) ordination plots showing the ecological and environmental (dis)similarity between and within nine communities on Cobb Seamount. NMDS plots were generated from Bray-Curtis similarity matrices of the faunal data for (A) all nine communities, (B) Communities 1 to 6, and (C) Communities 7 to 9. Similarly, NMDS plots were generated from Euclidian similarity matrices of the environmental data for (D) all nine communities, (E) Communities 1 to 6, and (F) Communities 7 to 9. Faunal data were for the presence-absence of 74 taxa, while environmental data include depth, slope, and small- and large-scale rugosity. Each community is represented by a different symbol and encircled by 95% similarity ellipses. The NMDS axes are unique for each plot.

NMDS ordination plots were used to illustrate the ecological and environmental (dis)similarity between and within communities identified by the cluster analysis (Fig 5). The primary ordination of the faunal similarity matrix (Fig 5A) preserved the original dissimilarities in the reduced number of dimensions of the plot (Shepard plot regressions: non-metric fit, R^2^ = 0.995 and linear fit, R^2^ = 0.991; stress = 0.067). Two tight, well-separated aggregations of samples indicated the high-level of dissimilarity between Communities 1 to 6 and 7 to 9 (those images taken by ROV and AUV, respectively). The two secondary ordinations of the faunal matrices for Communities 1 to 6 and 7 to 9 (Fig 5B and 5C, respectively) also preserved the original dissimilarities in the reduced number of dimensions (both Shepard plot regressions: non-metric fit, R^2^ ≥ 0.978 and linear fit, R^2^ ≥ 0.888; stress = 0.154 and 0.067). On the ordination plot of samples for Communities 1 to 6, similarity ellipses had less overlap, although all overlapped with at least one other aggregation of samples. The degree of separation varied between community samples, for example, Communities 5 and 6 were completely separate from Communities 1 and 2, while Community 3 samples had substantial overlap with samples for Community 5 and, to a lesser extent, Community 2. On the ordination plot for Communities 7 to 9, there was substantial overlap of the 95% similarity ellipses owing largely to the spread of samples for Community 8. The (dis)similarity illustrated by the NMDS was consistent, as expected, with values for number of unique taxa. For example, aggregations of samples for Communities 1 to 6 have less overlap between them than sample aggregations for Communities 7 to 9 (Fig 5B and 5C), with the former having a higher number of unique taxa (species turnover) between pairs of communities (Table 2).

The ordination of the environmental similarity matrix (Fig 5D) preserved the original dissimilarities in the reduced number of dimensions (Shepard plot regressions: non-metric fit, R^2^ = 0.999 and linear fit, R^2^ = 0.998; stress = 0.024). Like the faunal data ordination, the similarity ellipses around the samples of Communities 1 to 6 and 7 to 9 were separated into two main aggregations. The two secondary ordinations of the similarity matrices for environmental data from the locations for Communities 1 to 6 and 7 to 9 (Fig 5E and 5F, respectively) also preserved the original dissimilarities in the reduced number of dimensions (both Shepard plot regressions: non-metric fit, R^2^ ≥ 0.999 and linear fit, R^2^ ≥ 0.998; stress = 0.026 and 0.033). Unlike the faunal ordination, it was harder to distinguish sample aggregations from different communities. The similarity ellipse for Community 8 samples largely encompassed those for Communities 7 and 9. Sample aggregations for Communities 2 to 6 all overlapped with aggregations for at least one other community, but sample aggregations for Communities 4, 5 and 6 were separate from that for Community 2. Despite having the fewest image samples, the aggregation for Community 1 had the largest spread and was completely separate from aggregations for other communities.

A comparison of the primary faunal and environmental ordinations showed the degree of concordance (i.e., multidimensional shape similarity) between the two matrices is greater than expected given random inter-matrix associations (Procrustes analysis and PROTEST: *n* = 1464, m^2^ = 0.651, r^2^ = 0.591, *p* = 0.001).

### Community Distribution Modelling

**Fig 6:**
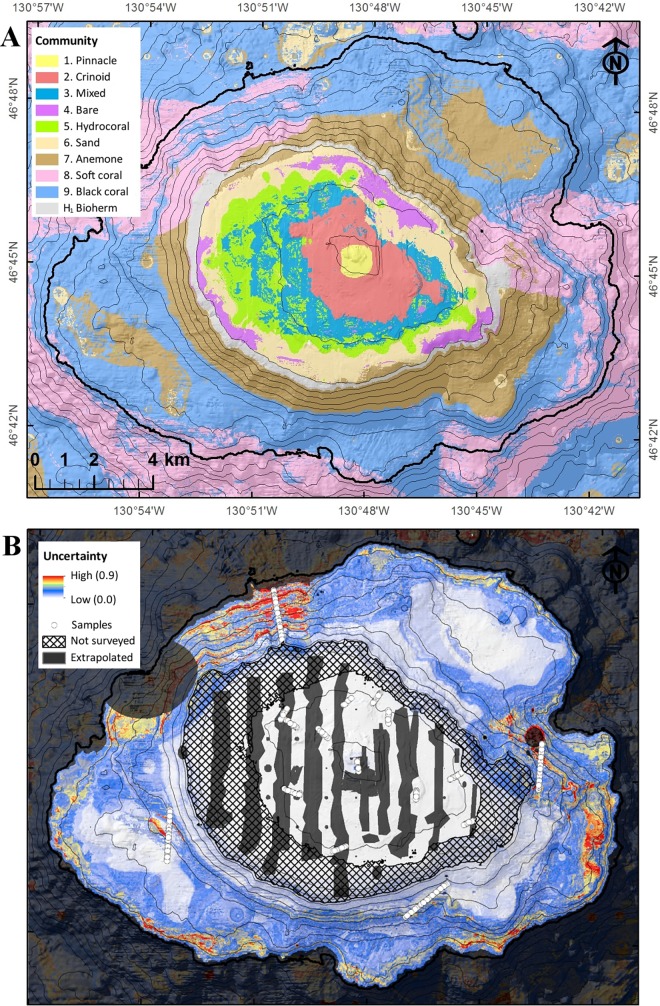
Outputs from the Random Forest modelling. (A) The predicted distributions of the nine communities, plus the hypothesized *Lophelia pertusa* bioherm community, and (B) the degree of model uncertainty. Model resolution is 20 by 20 m, thin black lines represent 100 m depth contours, the thick black line represents 1200 m (the approximate depth limit of the image surveys). (A) Each community is represented by a different colour, (B) white circles represent image locations, hatching represents the depth gap not surveyed (211 to 472 m), and dark gray shading represents extrapolated areas (i.e., areas where one or more environmental variable is beyond the sampled range).

The spatial distributions of the nine communities identified on Cobb Seamount, predicted by the Random Forest model, are shown in Fig 6A, and the descriptive statistics for the distributions are summarized in Table 4. Each predicted community distribution was a spatially cohesive, complete or partial band encircling the seamount. According to the model, the **Pinnacle** Community (1) was the smallest (<1 km^2^), shallowest community (<90 m depth) on Cobb Seamount. Down slope, the central summit was completely encircled by a wide band of the **Crinoid** Community (2; <180 m depth), followed by a band of the **Mixed** Community (3; <225 m depth). At its deeper limit, the Mixed Community was predicted to have a patchy transition into a narrow band of the **Hydrocoral** Community (5; approx. 200 to 250 m depth). The edge of the summit was predicted to be occupied by patches of the **Bare** Community (4) and a wide band of the **Sand** Community (6). Although not a model output, it is hypothesized that the summit ridge was encircled by a narrow band of an unsampled community typified by *Lophelia pertusa* bioherms (see Discussion). After the summit break (at 350 m), a band of the **Anemone** Community (7) was predicted to extend down slope to 750 m depth, although patches of the **Soft coral** Community (8) were predicted to occupy the steeper and more rugose areas of the shallow flanks (illustrated by contour lines in Fig 6A). The Soft coral Community was predicted to cover the largest depth range (350 to 1200 m), while the **Black coral** Community (9) was predicted to be the most extensive and deepest community on Cobb Seamount (33% of the total area >1200 m depth; depth range: 750 to 1200 m).

**Table 4:** A summary of the distributional and environmental characteristics of ten communities on Cobb Seamount based on a predicted distribution model (Fig 6A), and a hypothesized unsampled *Lophelia pertusa* Bioherm community.

Community number and name		Location	Spatial pattern	Environmental characteristic(s)	Depth (m)		Area		Width (km)	Model error (%)
					Min.	Max.	(km^2^)	(%)		
1	Pinnacle	Summit (pinnacle)	Band	Shallowest, euphotic zone	35	90	1.6	1	0.5	0
2	Crinoid	Summit	Band	Extends to wave-base limit	90	180	11.7	5	1.5	6
3	Mixed	Summit	Band	At euphotic zone limit	180	225	11.6	5	1	21
4	Bare	Summit	Patches	Patches of boulders & sand	275	350	7.2	3	0.5	48
5	Hydrocoral	Summit	Patches	Coral rumble as substrate	200	250	11.8	6	0.5	43
6	Sand	Summit	Band	Flat and sandy	250	350	18.4	9	0.75	5
H_1_	Bioherm	Ridge	Band	Rocky outcrops	300	360	-	-	-	-
7	Anemone	Flank	Band & patches	Deep and flat	350	750	49.6	23	1.5	25
8	Soft coral	Flank	Patches	Deep and rugose	350	>1200	30.3	14	2–3	33
9	Black coral	Flank	Band	Deepest	350	>1200	70.4	33	2–3	22

According to the mean decrease in accuracy, the most important predictor in the seamount community distribution model was depth (86% decrease in model accuracy if removed), followed by large-scale rugosity (60%), small-scale rugosity (56%), and slope (30%). The model reliability and limitations were high and low, respectively: AUC from 10-fold cross-validation = 0.9, and OOB error rate = 21%. The most common error was an incorrect prediction of an adjacent community; for example, the Anemone and Black coral Communities (7 and 8) were sometimes incorrectly predicted as the other. The lowest accuracy was between the predictions for the Mixed, Bare, and Hydrocoral Communities (3, 4, and 5; Table 4). In contrast, the Pinnacle Community (1) predictions were 100% accurate for both community presence and absence.

Using a bootstrap approach, confidence intervals were generated to represent the spatial uncertainty of the community distribution model (Fig 6B). There was no pattern between the proximity of the image locations and degree of uncertainty, but rather uncertainty increased with increasing depth and was particularly high on the northern and eastern flanks of the seamount. The distribution of the communities in some areas was extrapolated when either no environmental data were collected or both faunal and environmental data were not collected (hatched and dark gray shaded areas in Fig 6B), and therefore the uncertainty in these areas is unknown.

## Discussion

### What is the community structure on the shallow seamount?

The benthic mega-epifauna on Cobb Seamount have a discernible community structure. The cluster analyses identified nine distinct communities above 1200 m depth. This study is the first to quantitatively resolve this many communities on Cobb Seamount, likely in part because of the spatial coverage of the survey and the high-resolution image samples. The analyses further indicated the nine communities divide into two non-overlapping large-scale community groups: Communities 1 to 6 and 7 to 9 or the summit and flank. It must be remembered that this obvious distinction between the summit and flank community groups, which we believe to be real, is confounded by the depth operation range of the two methods used to obtain the images on which the analysis was based. However, a similar image-based study of two seamounts in the Indian Ocean also observed this marked distinction between summit and flank communities [41].

Previous surveys of Cobb Seamount have differentiated fewer communities, but these studies were restricted to qualitative descriptions at shallower depths (e.g., observations of four communities above 700 m [11]). Fewer communities have also been resolved on other Northeast Pacific seamounts, but these seamounts are substantially deeper than Cobb Seamount, and the studies surveyed larger areas per sample (e.g., Davidson Seamount [7]). On shallow seamounts, over similar depth ranges, comparable numbers of mega-epifaunal communities have been observed elsewhere in the world. On a Northeast Atlantic seamount, between 30 and 230 m depth, four depth-related communities were resolved [28]. In a similar manner, the present study identified that, between 34 to 210 m depth, four significantly different depth ranges were occupied by six communities (Communities 4 to 6 occupied the same range). In the Tyrrhenian Sea, on a shallow seamount, three communities were observed between 60 and 100 m depth, two of which occurred within the same depth range (70 to 100 m depth) but occupied different sides (aspects) of the seamount [42]. The present study also resolved two depth-related zones above 100 m but, in contrast, only two communities were resolved as occupying these zones.

### How do the seamount communities differ?

All taxa observed on Cobb Seamount are commonly found on the North American coast. There was no recorded endemism on the seamount [20]. Communities were primarily differentiated by the presence and absence of cold-water corals and sponges, macroalgae, and crustose coralline algae (cf. previous studies [7, 28]). Many species of these taxa provide biological structure and/or are primary producers. Although species may co-occur owing to similar environmental requirements, structural taxa and primary producers are commonly considered foundation species because they influence community composition through associations.

Biological structures alter available niches by modifying the surrounding conditions (e.g., flow regime and sediment composition) and creating structural habitat heterogeneity (e.g., substrate for attachment, shelter, feeding or parasitism) [43]. The complex biological structures of erect cold-water corals and sponges has been shown to attract mobile invertebrates and fish on other shallow seamounts [44, 45]. On Cobb Seamount, corals and sponges were observed as gardens (dense aggregations) or as solitary individuals, although some reef-forming taxa were observed (e.g., *Farrea occa* and *L*. pertusa [46, 47]). The *S*. *helvomaculatus* rockfish was only present on Cobb Seamount in communities with dense *Stylaster* spp. hydrocorals (Communities 4 and 5), and is known to associate with corals over bare substratum [48]. Deep-sea Chirostylidae spp. squat lobsters were most frequent on Cobb Seamount in coral and sponge prominent communities (Communities 8 and 9), and are known to associate with structural complexity [49]. In contrast, *Sebastolobus* spp. thornyheads were most frequent in the deep community that also lacked corals and sponges (Community 7; rare in coral- and sponge-dominated Communities 8 and 9), and have been shown to disassociate with structural complexity [48]. Cold-water corals and sponges are not the only taxa observed on Cobb Seamount that are known to provide biological structure. The *A*. *loveni* brittle star was only present on Cobb Seamount in shallow communities with the *H*. *willemoesi* sea whip (Communities 4 and 6), and is known to perch on Pennatulacea for enhanced feeding [50]. The highest frequency of fish on Cobb Seamount occurred in the *F*. *serratissima* crinoid prominent community (Community 2), and *F*. *serratissima* is known to form dense aggregations that enhance fish habitat [51].

Primary producers exert a bottom-up control on community composition through trophic dynamics [52]. The coocurrance of macro- and crustose coralline algae and associated grazers, detritivores, and structure-seeking species have been described on shallow seamounts world-wide (e.g., NE Pacific [53]; NE Atlantic [28, 54]; SE Atlantic [55]; SW Atlantic [56]; Mediterranean [42, 57]). The *Calliostoma* spp. topsnails, *L*. *rugatus* chiton, and *M*. *franciscanus* sea urchin were only present on Cobb Seamount in the community with both Corallinales spp. and *D*. *viridis* (Community 1), and are all algae grazers or detritivores [58, 59]. On Cobb Seamount, *D*. *viridis* formed dense canopies, obscuring the FOV and limiting the survey of the pinnacle. On other shallow seamounts, macroalgae has been shown to support high local macrofaunal richness, abundance, biomass, and diversity [57]. It is likely there were more macro-epifauna associated with the benthic primary producers on the Cobb Seamount summit than presented here.

It is apparent that Cobb Seamount's communities were further differentiated by taxa known to relate to the aforementioned foundation taxa. The compositions of the nine communities on Cobb Seamount corroborate that corals and sponges exhibit relatively strong influences on associated seamount communities, and that on shallow seamounts, algae support local secondary production and macroalgae provide significant biological structure [4, 5, 28, 56, 60, 61].

### Does seamount community structure correspond to seafloor environmental patterns?

Our results indicate that the community structure on Cobb Seamount corresponds to seafloor environmental patterns related to rugosity, slope, and substrate, but it is primarily depth-stratified. Vertical zonation, reflecting ecologically significant environmental gradients correlated with depth, is common on seamounts [4, 7, 28, 62, 63] and has been previously reported on Cobb Seamount above 180 m [18] and 700 m depth [11]. In the present study, the nine communities were distributed within six significantly different depth ranges. These depth differences mirrored the ecological distances between communities; the rank order of communities from shallowest to deepest was the same as the rank order of the assigned clusters despite the data input having been randomized. There was a notable trend of the shallowest communities occupying the narrowest bands, likely owing to pronounced environmental gradient changes at shallow depths, such as primary production in the euphotic zone (both benthic and phytoplankton) and wave base (i.e., hydrodynamic forces created by water waves). On Cobb Seamount, the two shallowest communities (1 and 2) were differentiated by the distribution of brown *D*. *viridis* algae (Ochrophyta) and red Corallinales spp. algae (Rhodophyta), which have different depth limits due to light requirements for photosynthesis [11, 20, 64]. Community 2 was also typified by the *F*. *serratissima* crinoid. *F*. *serratissima* only inhabits areas with specific gentle flow [65], and the highest frequency of *F*. *serratissima* corresponded with the local wave base limit at ca. 150 m [11]. Community 3 was defined by the overlap of two foundation taxa (Corallinales spp. and *Stylaster* spp.) and it had a narrow depth range, high taxa richness and low number of unique taxa—all of which are qualities of an ecotone (i.e., an environmental transition zone where two communities meet and integrate [66]). The depth distribution of this ecotone corresponded with—and was likely driven by—the sharp depth gradient of the local photic zone limit at ca. 180 m [11]. Similar depth-related community patterns have been reported for other shallow seamounts (e.g., NE Atlantic [28, 67]; SE Atlantic [56]).

Other depth-related gradients include pH, pressure, and temperature, all of which are factors that can constrain species' distributions far below the euphotic zone and wave base. For example, vertical zonation of coral assemblages on a Hawaiian slope (<530 m depth) [68] and benthic communities on Tasmanian seamounts (<4 km depth) [63]. On Cobb Seamount, the two deepest communities (8 and 9) were differentiated by the distribution of cold-water corals and sponges. On Cobb Seamount, the highest frequency of Antipatharia spp. (black) occurred in the deepest community (Community 9; av. depth: 857 m) [11], while the ecologically similar Alcyonacea (soft) corals and Hexactinellida (glass) sponges were most frequent in the adjacent shallower community (Community 8; av. depth: 728 m). Antipatharia spp. corals are known to be more abundant with depth because of interspecific competition with shallower species [69].

The factors that vary with depth, although a prominent influence, did not account for all community structure on Cobb Seamount. Community variability also corresponded with patterns in environmental variables that were not correlated to depth. Several communities with overlapping depth ranges, but distinctly different species compositions were differentiated by large-scale rugosity, small-scale rugosity, and slope (in order of decreasing importance from the Random Forest model). For example, Communities 7 and 8 occupied the same depth range (av. depth: 740 and 728 m) but Community 8 occured on harder substrates in steeper, more rugose areas (hard substrate categories: 76 and 34%; av. slope: 19 and 16°; av. small-scale rugosity: 1.09 and 1.07; av. large-scale rugosity: 1.07 and 1.06; for Communities 8 and 7, respectively). The cold-water corals and sponges of Community 8 require hard substrate for anchorage and specific flow regimes to filter-feed [45, 70, 71]; slope and rugosity are proxies for both requirements [72, 73]. It is likely the lava cones and steep lava terrace edges that cover the flanks of the seamount [12] created the environmental conditions necessary for the Soft coral Community. In contrast, it is likely the well-developed, flat-topped lava terraces that cover the flanks of the seamount [12] created the environmental conditions required by the Anemone Community (unconsolidated substratum in areas that are less steep and less rugose). Although the rugosity metric used was confounded with slope in the study, the distribution of cold-water corals off Hawaii was primarily attributed to depth, with slope and rugosity also identified as important variables [68].

The overall slope of Cobb Seamount was seven times steeper than the adjacent North American continental slope (measured between 34 and 1154 m; present study). The combination of the vertical zonation of narrow communities and the steep slopes of Cobb Seamount supports the hypothesis that high species turnover between vertically distributed communities ultimately produces the relatively high total biodiversity observed on seamounts [7]. It is notable that on Cobb Seamount, like on other Northeast Pacific seamounts, there was a unimodal relationship between the number of unique taxa (species turnover) and depth [62].

Seafloor substrate influences benthic community structure, both directly and indirectly (e.g., larvae settlement, anchorage, shelter, modified hydrodynamics). Although continuous data for substratum type were not available, categorical data from in-situ observations indicated most communities occurred on specific substrate types. On occasion, substrate type was the only environmental variable that differed between the location of communities. For example, the changes in substrate composition between weakly defined steps and terraces of the summit plateau were the result of varying origins, volcanic activity, and a complex subsidence history [11], and these substrate types were the only environmental difference detected between locations where Communities 4, 5, and 6 occurred. Subsequent ecological studies similar to Cobb Seamount would greatly benefit from the collection of multibeam backscatter, continuous data which can be used as a proxy for substratum type [74].

### What is the capability of using remotely-sensed data to predict the spatial distribution of the seamount communities?

The predicted community distribution patterns were consistent with the vertical zonation commonly found on seamounts [4], which is not surprising since the Random Forest model identified depth as the most important predictor of community distribution, followed by large-scale rugosity, small-scale rugosity, and slope. These four environmental variables have all been proven useful in predicting the distribution of seamount fauna, depth and slope more commonly than rugosity (e.g., cold-water corals and sponges [75–78]). It should be noted that, when rugosity has been included in models, the metrics used were often inadvertently coupled, and so confounded, with slope [31]. That said, the relatively high predictive power of large-scale rugosity decoupled from slope is supported by findings of a multi-scale ACR rugosity analysis on a Northeast Pacific submarine bank [79].

The Cobb Seamount community distribution model is an example of environmental surrogates successfully been used as faunal proxies on a seamount [6]. The overall performance of the model was very good, with a 21% estimated error rate and AUC score of 0.9. Model errors varied between communities; Community 1 was accurately predicted every time, while Community 4 was closer to 50% (where a 89% error rate would be random or by chance). The highest errors were incurred between pairs of communities that, in the environmental analyses, were only differentiated by substrate types. It would be expected that the accuracy of the model would be improved by the inclusion of spatial substrate data (e.g., multibeam backscatter). It should also be noted that Cobb Seamount is commercially fished and bottom fishing impacts were observed during the 2012 survey (e.g., cold-water corals and sponges entangled with gear and/or toppled over) [19, 20] and including a metric of fishing intensity may also improve the accuracy of the model. This model has already successfully been used by Fisheries and Oceans Canada to predict the spatial distribution of benthic communities on other Northeast Pacific seamounts, to help plan survey designs (e.g., Bowie Seamount; cruise report in prep).

The model accuracy would likely also be higher if there was less variance in the sample numbers between communities. The number of images per community varied by an order of magnitude; however, the proportion of samples per area covered by the community was the same. According to previous surveys of the seamount pinnacle, it is only 880 m long by 577 m wide by 75 m tall [17, 18]. The model predicted a similar small area for Community 1 (1.6 km^2^, where *n* = 11) while it predicted Community 9 covers nearly 50 times the area (70.4 km^2^, where *n* = 489). With this large difference in scale, sampling each community equally would be impractical. Although the best survey design for sampling to build a model would include higher spatial coverage with either random or gridded surveys, this is not usually an option when working in remote, deep-sea locations using submersibles. The selected study scale and resolution can also have significant effects on a model output, because it is likely that a difference to either would have resulted in discerning slightly different communities.

The uncertainty map provided a guide as to what areas of the predicted community distribution map are the most and least reliable. Model predictions from the high-uncertainty or extrapolated areas should be assessed and used with limitations considered. The shallow predictions tended to be the most certain while uncertainty increased with increasing depth until 1200 m, after which uncertainty was still variable but the predictions were extrapolated (i.e, beyond the sampled depth range). In addition, there were two circular extrapolated areas on the deep flanks of the seamount: a large area on the northwestern slope and a small area on the eastern slope. Although these areas were within the depth range sampled, that they were identified as extrapolated indicates at least one of the other environmental variables was outside the sampled ranges (i.e., a higher rugosity and/or slope value than what was sampled). The extrapolated areas on the summit (where no bathymetric data were available) are a function (kriging interpolation) of the surrounding, similar environments that were well sampled, and the predictions should be considered fairly reliable.

Owing to technical issues, it was not possible to survey between 211 and 472 m depth. Predictions of community distribution were modelled into this area, and not extrapolated because these depths fell within the surveyed range. However, there is subsequent supporting ground-truthed evidence that predictions in this depth range are at least partly supported. The model predicted Community 8 extended up the slope to 350 m, 100 m shallower than recorded in a visual survey. During dives that were not included in this study, observations were made of high densities of the defining taxa of Community 8 at 375 m (the Alyconacea, Primnoidea corals) [19]. At its deepest, Community 4 was observed at 210 m by the visual surveys but the model predicted it extended another 140 m, to 350 m depth. Community 4 occurred exclusively on sand dominant substrates and sediment scoops from 310 m indicate this area, unsampled by this study, was an ancient sandy beach (it is thought to have originated intertidally when sea-level was lower) [11].

There is also evidence that the unsampled depth gap in the surveys resulted in a community going undetected. On Cobb Seamount, the reef-building coral *L*. *pertusa* has been described as abundant between 300 and 360 m depth, with large bioherms occurring on pillowed dykes on the eastern shoulder of the seamount (Tunnicliffe, 1982, unpublished raw data [11]). As with the Primnoidea spp. observations made during dives that were not included in this study, observations were made of high densities of *L*. *pertusa* at 254 m depth [20]. *L*. *pertusa* was also observed as shallow as 162 m depth but it was rare (<1% of images) and therefore not included in the community analysis. If the entire depth gradient was sampled, it is likely that a *L*. *pertusa* defined community would have been found between the two non-overlapping groups of communities identified by the community analysis (Communities 1 to 6 and 7 to 9). It follows then that the predicted spatial extent of Community 7 would be reduced by the hypothesised *L*. *pertusa* Bioherm Community.

## Conclusions

Nine benthic mega-epifaunal communities were identified on Cobb Seamount above ~1200 m based on the distribution of 74 taxa from 11 phyla, with possibly a tenth community thought to exist in unsampled space between 211 and 472 m depth. Each community was typified by foundation taxa considered to provide significant biological structure and/or taxa that are primary producers—the majority of which are potential VME indicator taxa (e.g., cold-water corals and sponges). The benthic community structure on the shallow seamount corresponded to observed environmental variability in depth, seafloor rugosity, substratum, and slope. Depth was the strongest environmental proxy for the community-structuring processes, and communities were generally distributed as bands encircling the seamount, either on the summit or on the flanks. The variability in the distribution of communities with overlapping depth ranges corresponded to patterns of rugosity (at two scales; where the large-scale outperformed the small-scale metric), substrate type and, to a lesser degree, slope. The environmental metrics derived from the ship-based multibeam bathymetry were successfully used as surrogates, to produce accurate and reliable Random Forest model predictions of the distribution of benthic mega-epifaunal communities over a 220 km^2^ region, the top 1200 m of Cobb Seamount, at a resolution of 20 m x 20 m. A map of this size and resolution should prove helpful for ecosystem-based management and impact assessment of a seamount [6]. This study also supports the viability of using relatively easy-to-survey structural taxa (e.g., cold-water corals and sponges) as proxies for predicting the distributions of communities or other individual species.

The findings on the structure, diversity and distribution of benthic mega-epifauna communities on Cobb Seamount offer empirical evidence supporting and refuting several prevailing paradigms in seamount ecology [5]. The relatively large number of narrow, banded communities reflects high species turnover, and supports the paradigm that seamounts are ‘hotspots’ of biodiversity [5]. That most communities were typified by a potential VME indicator taxa, and that fishing does occur on Cobb Seamount and does impact the benthos [19], supports the paradigm that seamount communities are at risk to disturbance from bottom fishing [5]. That most communities were typified by at least one organism known to be highly susceptible to ocean acidification (e.g., crustose coralline algae and the aragonitic corals *Stylaster* spp. and *L*. *pertusa* [56, 80, 81]), and that Cobb Seamount may serve as an area of oceanographic stability [21, 81], supports the paradigm that seamounts are potential refugia for biota from marine climate change [5]. That all taxa observed during the visual survey are commonly found on the North American coast, refutes the paradigm that seamounts have high levels of endemism (a paradigm already in dispute for seamounts in the Northeast Pacific [5, 82]).
